# Exercise hyperthermia induces greater changes in gastrointestinal permeability than equivalent passive hyperthermia

**DOI:** 10.14814/phy2.14945

**Published:** 2021-08-19

**Authors:** Edward Walter, Peter W Watt, Oliver R. Gibson, Ashley G. B. Wilmott, Dominic Mitchell, Robert Moreton, Neil S. Maxwell

**Affiliations:** ^1^ Department of Intensive Care Royal Surrey County Hospital Guildford UK; ^2^ Environmental Extremes Lab, Sport and Exercise Science and Medicine Research and Enterprise Group University of Brighton Eastbourne East Sussex UK; ^3^ Centre for Human Performance, Exercise and Rehabilitation (CHPER) Division of Sport, Health and Exercise Sciences College of Health, Medicine, and Life Sciences Brunel University London Uxbridge UK; ^4^ Cambridge Centre for Sport and Exercise Sciences (CCSES) School of Psychology and Sport Science Anglia Ruskin University Cambridge UK

**Keywords:** cardiovascular, exercise, gastrointestinal, heat illness, hyperthermia, permeability

## Abstract

Hyperthermia and exertional heat illness increase gastrointestinal (GI) permeability, although whether the latter is only via hyperthermia is unclear. The aim of this pilot study was to determine whether different changes in GI permeability, characterized by an increased plasma lactulose:rhamnose concentration ratio ([L:R]), occurred in exercise hyperthermia in comparison to equivalent passive hyperthermia. Six healthy adult male participants (age 25 ± 5 years, mass 77.0 ± 6.7 kg, height 181 ± 6 cm, peak oxygen uptake [$\overset{\cdot }{V}O_{2\text{peak}}$] 48 ± 8 ml.kg^−1^.min^−1^) underwent exercise under hot conditions (Ex‐Heat) and passive heating during hot water immersion (HWI). Heart rate (HR), rectal temperature (T_CORE_), rating of perceived exertion (RPE), and whole‐body sweat loss (WBSL) were recorded throughout the trials. The L:R ratio, peak HR, change in HR, and change in RPE were higher in Ex‐Heat than HWI, despite no differences in trial duration, peak core temperature or WBSL. L:R was strongly correlated (*p* < 0.05) with HR peak (*r* = 0.626) and change in HR (*r* = 0.615) but no other variable. The greater L:R in Ex‐Heat, despite equal T_CORE_ responses to HWI, indicates that increased cardiovascular strain occurred during exercise, and exacerbates hyperthermia‐induced GI permeability at the same absolute temperature.

## Graphical abstract

Hyperthermia and exertional heat illness increase gastrointestinal (GI) permeability, although whether the latter is only via hyperthermia is unclear. In this study, GI permeability, characterised characterized by an increased plasma lactulose:rhamnose concentration ratio, was significantly higher in exercise hyperthermia in comparison to equivalent passive hyperthermia.

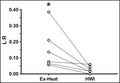

## 1 INTRODUCTION

Heat illness, whether due to exertional activity (exertional heat illness [EHI]) or passive heat gain, for example, in heatwaves, carries a significant risk of morbidity and mortality. Heatstroke, the most extreme form of heat illness, is associated with a risk of multi‐organ failure, including renal, liver, and brain dysfunction (Walter et al., 2016), and a mortality rate in classical heatstroke of up to 64% (Pease et al., 2009). Exertional heatstroke (EHS) is among the leading causes of sudden death in athletes, with the number of sports‐related EHS deaths in the United States estimated to have doubled since 1975 (Nichols, 2014). The tissue and organ dysfunction associated with heat illness appears to be related to an increase in gastrointestinal (GI) permeability, which may allow the translocation of intestinal bacteria or endotoxins into the systemic circulation (Lim, 2018

Hyperthermia increases GI permeability, though it remains equivocal whether this is a result of the direct effect of temperature on the intestinal epithelium, or whether competing demands for finite cardiac output increases between central and peripheral circulation creates local ischemia, which, in turn, reduces paracellular resistance to heat stress (Hall et al., 2001). Both exercise (March et al., 2017; Smetanka et al., 1999) and heat strain in vitro (Dokladny et al., 2006; Hall et al., 2001; Koch et al., 2019) damage gut wall integrity. An in vivo animal study by Lambert et al. (2002) provides histological and functional evidence of an increased loss of gut wall integrity during increasing levels of passive heat stress. Exercise‐induced heat stress has also been shown to disrupt intestinal epithelial cell tight junction proteins, such as occludins and claudins, resulting in increased permeability (Zuhl, 2014). According to a systematic review, the magnitude of exercise‐induced hyperthermia is directly associated with the increase in intestinal permeability (Pires et al., 2017). The authors identified that during exercise heat stress, both core temperature and intestinal permeability (largely characterized by lactulose:rhamnose concentration ratio) increase with a positive and strong correlation observed between the two parameters. Further to this, the review suggests that a core temperature exceeding 39℃ is always associated with augmented permeability (Pires et al., 2017). Data from others have suggested that it is equivocal whether heat stress or exercise is the primary determinant of increased GI permeability in EHI (Pires et al., 2017; Shing et al., 2014). Further to this, a recent study on the gut biomarker intestinal fatty acid‐binding protein (I‐FABP) suggests that after endurance exercise, I‐FABP is raised (+130%) compared with pre‐race levels, despite no change in core temperature, but is significantly higher (up to a further 10‐fold) after EHI, and persists for several hours despite cessation of exercise and core temperature returning to baseline (Walter et al., 2021). Determining the independent and combined effects of heat stress and exercise on GI permeability may further our understanding of the pathophysiology of EHI, in particular, the subsequent endotoxemia and inflammatory response, which may support the development of preventative and/or therapeutic interventions.

The aim of this pilot study was to determine whether GI permeability, characterized by plasma lactulose:rhamnose concentration ratio (L:R), differed between exercise hyperthermia in comparison to equivalent passive hyperthermia. It was hypothesised that exercise hyperthermia would increase GI permeability to a greater extent than passive hyperthermia at an equivalent core temperature, in association with a greater elevation in cardiovascular strain.

## 2 MATERIALS AND METHODS

### 2.1 Ethical approval and participants

The study was approved by the Institutional Research and Ethics Committee (ethics number SSCREC18‐12) and was conducted in accordance with the principles of the Declaration of Helsinki (2013). Six healthy adult male participants (age 25 ± 5 years, mass 77.0 ± 6.7 kg, height 181 ± 6 cm, peak oxygen uptake [$\overset{\cdot }{V}O_{2\text{peak}}$] 48 ± 8 ml.kg^−1^.min^−1^) provided written informed consent. Participants were excluded if they had previously had severe heat‐related illness, received anti‐inflammatory drugs, steroids or antibiotics in the preceding 3 months, or had consumed ergogenic aids or supplements in the 48 h prior to enrolment in the study or at any time during the study period itself. Participants were also excluded if they had any known pre‐existing GI or renal dysfunction.

### 2.2 Experimental design

Participants completed a preliminary trial consisting of baseline measurements and a graded exercise test (GXT) before the two main experimental trials: (i) exercising in hot conditions (Ex‐Heat) and (ii) passive heating during hot water immersion (HWI). Each of the main trials was separated by at least 7 days and was completed from April to July, at the same time of day (09:00). Participants refrained from strenuous exercise, caffeine, alcohol, fish, and dairy products for at least 24 h before all trials and replicated food intake the day prior to each trial. Participants consumed 3–5 ml.kg^−1^ of water 2 h before each trial to ensure adequate euhydration, as determined by urine specific gravity ≤1.020 (hand refractometer, Atago) and osmolality ≤700 mOsm.kg^−1^ (Osmocheck, Vitech Scientific Ltd.; Sawka et al., 2007).

### 2.3 Preliminary trial

During the preliminary trial, anthropometric data for height (SECA stadiometer) and body mass (Adam GFK 150 Body Scales, Adam Equipment Inc.) were recorded. The two‐part GXT was adapted from James et al. (2015) and completed on a motorized treadmill (Woodway ELG2). The GXT was conducted in a controlled environmental chamber (TISS) set to 40℃ and 40% relative humidity (RH), to determine lactate threshold and $\overset{\cdot }{V}O_{2\text{peak}}$. The submaximal phase of the GXT commenced between 8 and 10 km.h^−1^ (1% gradient throughout). Each stage lasted 4 min (3 min of exercise and 1 min of passive rest), after which, capillary blood was sampled to assess blood lactate concentration ([La]). After each stage, the treadmill speed increased 1 km.h^−1^ until an exponential increase in [La] was observed. Following 15 min of rest, participants completed the maximal phase which began at a corresponding speed of 2 km.h^−1^ below the final submaximal stage, then subsequently increased by a 1% gradient per minute until volitional exhaustion. Heart rate (HR), rating of perceived exertion (RPE; Borg, 1982), and expired air were recorded in the final 45 s of each stage.

### 2.4 Main experimental trials

Following baseline blood sampling and instrumentation, participants ran at 9 km.h^−1^ (1% gradient), in 40℃ and 40% RH for the Ex‐Heat trial, whereas the HWI trial was performed in an immersion tank at 40℃ with water raised to the participant's sternum. Trials continued until core temperature (T_CORE_) reached 39.7℃ or volition necessitated cessation (zero cases). Following the completion of the trial, participants were immediately removed from the environmental chamber, with nude body mass recorded to estimate whole‐body sweat loss (WBSL). At 105 min after the commencement of exercise (90 min after consuming the disugar probe), a 10 ml blood sample was taken, after which, participants were permitted to consume fluid.

### 2.5 Assessments

#### 2.5.1 Physiological measures and instrumentation

A mid‐flow urine sample was used to determine hydration status and then nude body mass was recorded (±0.01 kg). Participants self‐inserted a thermistor (Henleys Medical Supplies; YSI Model 401) 10 cm past the anal sphincter to measure T_CORE_ and affixed a chest strap to measure HR (Polar F1), both at 10 min intervals and trial completion. WBSL was estimated for each trial from pre to post, towel‐dried nude body mass differences.

#### 2.5.2 Perceptual measures

Perceptual assessment using the RPE, thermal sensation (TS), and thermal comfort (TC) scales was collected during the main trials every 10 min following familiarization. RPE rates perceived exertion from 6 (*no exertion*) to 20 (*maximal exertion*). The TS (Toner et al., 1986) uses a scale between 0 (*unbearably cold*) and 8 (*unbearably hot*), and the TC (Zhang et al., 2004) between 0 (*very comfortable*) and 5 (*very uncomfortable*). The gut permeability symptom scale (GPSS; Wilson, 2017) is scaled between 0 (*no discomfort*) and 10 (*unbearable discomfort*). GPSS was measured immediately before and after each trial.

#### 2.5.3 Assessment of gastrointestinal permeability and sample analysis

GI permeability was assessed by the measurement of plasma lactulose and rhamnose, with the L:R subsequently calculated, as described by Pugh et al. (2017), based on a previously described technique (Fleming et al., 1996). Rhamnose (2 g; R3875, Sigma‐Aldrich) and 5 g of lactulose (PL 00030‐0175, Novartis) doses were measured (Scalix Pocket Balance, 0.01 g ATP instrumentation Ltd.), dissolved in hot water to a total volume of sugar dose solution of 50 ml, and allowed to cool to room temperature before being ingested at 15 min following the commencement of the trial.

For plasma extraction, a 10 ml venepuncture sample was collected from the antecubital fossa. The sample was then transferred into two 5‐ml tubes (ethylenediaminetetraacetic acid [EDTA] Sarstedt, Aktiengesellschaft and Co) and once clotted, centrifuged (Eppendorf 5702 R Centrifuge) for 10 min at 5000 rev.min^−1^. Plasma was aliquoted and stored in 1.5 ml capped microtubes (Western Laboratory Service) at −86℃ until analysis. Sample analysis was performed via high‐performance gas chromatography‐mass spectrometry on a Clarus 500D GCMS. Sample preparation was based on the method of Jansen et al. (1986); the sugars were derivatized to their trimethylsilyl compounds and mass selective analysis of m/z 204 for rhamnose and m/z 129 and 239 for lactulose quantified using the GCMS manufacturer‐supplied software.

### 2.6 Statistical analysis

Data were assessed and conformed to normality and sphericity prior to further statistical analysis. A two‐way ANOVA test was used to determine changes over time (pre and post) and between trials (Ex‐Heat and HWI). Bonferroni post hoc tests were used following the identification of a main or interaction effect. For pre–post and change data, parametric variables (L:R, trial duration, WBSL, T_CORE_, HR) were analysed using paired sample *t* tests, with non‐parametric variables (RPE, TS, TC, and GPSS), compared using Wilcoxon signed ranks. Pearson's correlations were performed between the L:R and all dependent variables pooled from Ex‐Heat and HWI trials. All normal data are reported as mean ± standard deviation (SD), and non‐normal data as median ± interquartile range (IQR), with statistical significance set at *p* ≤ 0.05.

## 3 RESULTS

A difference over time was identified for T_CORE_ (*f* = 1768.8, *p* < 0.001), HR (*f* = 1375.5, *p* < 0.001), RPE (*f* = 38.3, *p* = 0.002), TS (*f* = 141.8, *p* < 0.001), and TC (*f* = 489.6, *p* < 0.001), with no difference in GPSS (*f* = 4.153, *p* = 0.097). Only HR (*f* = 34.5, *p* = 0.002), RPE (*f* = 17.4, *p* = 0.009), TC (*f* = 37.5, *p* = 0.002), and GPSS (*f* = 35.7, *p* = 0.002) differed between trials, with no change in T_CORE_ (*f* = 0.03, *p* = 0.852) or TS (*f* = 0.1, *p* = 0.793). An interaction effect was identified for HR (*f* = 96.6, *p* < 0.001) and RPE (*f* = 17.4, *p* = 0.009) only (see Table 1).

**TABLE 1:** Table to show the lactulose:rhamnose ratio (L:R), trial duration, and recorded resting, peak, and change in physiological and perceptual variables during exercising in hot conditions (Ex‐Heat) and passive heating during hot water immersion (HWI) trials (*n* = 6)

	Ex‐Heat	HWI	Ex‐Heat – HWI difference	Pearson's correlation versus L:R
L:R	0.15 ± 0.13*	0.03 ± 0.02	+0.13 ± 0.12	—
Duration (min)	44.1 ± 7.8	56.0 ± 16.6	−11.9 ± 13.5	*r* = −0.502
WBSL (L)	1.5 ± 0.6	1.5 ± 0.9	0.0 ± 1.0	*r* = −0.320
T_CORE_ rest (℃)	37.04 ± 0.19	36.99 ± 0.28	+0.05 ± 0.26	*r* = 0.092
T_CORE_ peak (℃)	39.30 ± 0.20^^^	39.32 ± 0.19^^^	−0.02 ± 0.22	*r* = −0.396
∆T_CORE_ (℃)	2.26 ± 0.17	2.33 ± 0.19	−0.07 ± 0.24	*r* = −0.539
HR rest (b.min^−1^)	60 ± 9	62 ± 12	−2 ± 8	*r* = 0.015
HR peak (b.min^−1^)	168 ± 10* ^,^ ^^^	110 ± 11^^^	+58 ± 18	*r* = 0.626 ^#^
∆HR (b.min^−1^)	107 ± 9*	48 ± 9	+60 ± 15	*r* = 0.615 ^#^
RPE rest	6 ± 0	6 ± 0	0 ± 0	*r* = 0.000
RPE peak	16 ± 2* ^,^ ^^^	6 ± 5^^^	+13 ± 5	*r* = 0.407
∆RPE	10 ± 2*	0 ± 5	9 ± 5	*r* = 0.407
TS rest	4.0 ± 0.0	4.0 ± 0.0	0.0 ± 0.4	*r* = −0.073
TS peak	7.3 ± 1.3^^^	7.5 ± 0.8^^^	0.0 ± 0.6	*r* = 0.018
∆TS	2.8 ± 1.3	3.5 ± 0.8	−0.3 ± 0.7	*r* = 0.046
TC rest	1 ± 0	1 ± 0	0 ± 0	*r* = 0.000
TC peak	5 ± 1^^^	6 ± 1^^^	−1 ± 1	*r* = −0.341
∆TC	4 ± 1	5 ± 1	−1 ± 1	*r* = −0.341
GPSS rest	0 ± 1	1 ± 1	0 ± 2	*r* = −0.236
GPSS peak	3 ± 6*	1 ± 3	+3 ± 3	*r* = 0.250
∆GPSS	3 ± 6	0 ± 2	3 ± 5	*r* = 0.298

Note

Data are mean ± SD except RPE, TS, TC, and GPSS, reported as median ± IQR.

Abbreviations: ∆T_CORE_, change in core body temperature; GPSS, gut permeability symptom scale; HR, heart rate; RPE, rating of perceived exertion; TC, thermal comfort; T_CORE_, core body temperature; TS, thermal sensation; WBSL, whole‐body sweat loss.

* Significant difference from HWI (*p* < 0.05).

^ Significant difference overall from rest (*p* < 0.05).

# Significant relationship with L:R (*p* < 0.05).

**FIGURE 1:**
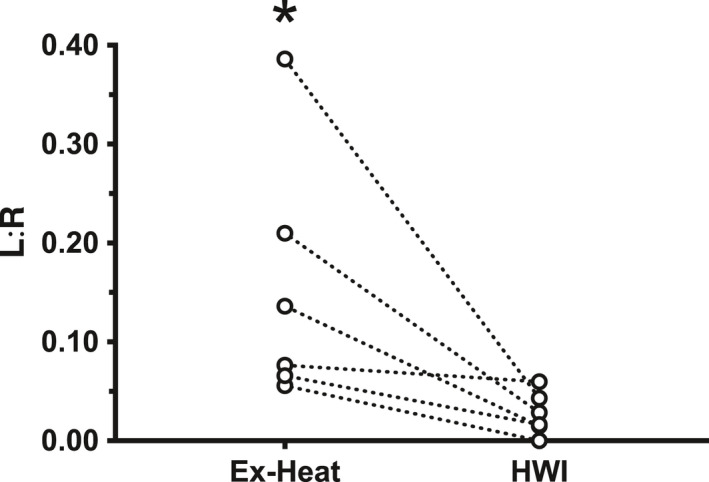
Lactulose:rhamnose ratio (L:R) for each participant during exercising in heat stress (Ex‐Heat) and passive heating during hot water immersion (HWI) trials. * denotes significant group mean difference compared with HWI

Differences between Ex‐Heat and HWI were observed for L:R (Figure 1; *t* = 2.595, *p* = 0.049), HR peak (*t* = 7.958, *p* = 0.010), change (∆) in HR (*t* = 9.830, *p* < 0.001), RPE (*Z* = 2.003, *p* = 0.045), and GPSS (*Z* = 1.997, *p* = 0.046). No differences (*p* > 0.05) were observed for any other variables (see Table 1). The L:R correlated with HR peak (*r* = 0.626, *p* = 0.029) and ∆HR (*r* = 0.615, *p* = 0.033).

## 4 DISCUSSION

In agreement with our experimental hypothesis, the post‐exercise L:R was greater in exercise hyperthermia (Ex‐Heat: +0.15 ± 0.13) than passive hyperthermia (HWI: +0.03 ± 0.02), despite no differences in T_CORE_ (both +2.3℃), WBSL (both +1.5 L; −1.9% body mass) or perceptual responses between these isothermic, iso‐duration trials. The L:R responses generally exceeded that previously reported for similar absolute T_CORE_ values (L:R +0.04–0.11 and T_CORE_ 38.0–39.6℃, respectively; Pires et al., 2017), although this may reflect blood rather than urinary sampling and the measurement of a single post‐trial sample, rather than pre–post change. The observation that for the same T_CORE_ (Ex‐Heat: 39.30 ± 0.20℃ vs. HWI: 39.32 ± 0.19℃), HR peak, and ∆HR was greater during Ex‐Heat compared with HWI, (Ex‐Heat vs. HWI; HR peak +58 ± 18, ∆HR +60 ± 15 b.min^−1^, respectively), coupled with the significant relationship between L:R and HR peak and ∆HR (*r* = 0.626 and 0.615, respectively), points to a possible role that cardiovascular strain may exacerbate GI permeability during exercise hyperthermia.

The increase in GI permeability observed with hyperthermia is likely to be multifactorial. Possible mechanisms include direct thermal damage to epithelial cells, and changes in splanchnic circulation leading to oxidative stress (Lambert et al., 2002). Pires et al. (2017) report a strong positive correlation (*r* = 0.793, *r* ^2^ = 0.629 *p* < 0.001) between T_CORE_ and urinary L:R; however, our findings for plasma L:R and change (*r* = −0.539, *p* = 0.070) or peak T_CORE_ (*r* = −0.396, *p* = 0.202) do not agree.

Exercise increases GI permeability more so than hyperthermia alone, yet it is unclear whether this is due to the aforementioned increased physiological strain or by additional mechanisms. Splanchnic blood flow falls by 20% at a T_CORE_ of 38℃ compared with 37℃, falling further with higher temperatures (Badoer, 2010). During exercise, blood flow has been shown to reduce in the superior mesenteric artery by 43% (Qamar & Read, 1987), and in the portal vein by 50% (Hayashi et al., 2012); however, in neither of these studies was the effect of temperature assessed. Our data and these mechanistic studies collectively point toward the competing demands for blood flow from the central and peripheral circulation during hyperthermia (González‐Alonso et al., 2008), induced by exercise‐heat stress, which serves as a greater stressor on the gut than equivalent hyperthermia via passive heat stress. The cardiovascular strain of exercise‐heat stress creates significant local (GI) ischemia, which in turn, reduces paracellular resistance to heat stress, ultimately leading to gut leakage (Hall et al., 2001).

Unsurprisingly, there was a higher RPE after exercise compared with before, and compared with after passive heating (+8 ± 5 [“*Very hard*” vs. “*Very light*”]). TS increased (both: “*Very hot*”) and TC decreased (both: “*Very uncomfortable*”) after exercise and passive heating, with no difference between the trials (−0.1 ± 0.6 and −1 ± 1, respectively). GPSS were higher after exercise than baseline, and higher than after HWI (+3 ± 3 [‘*Moderate discomfort’*]), which correlates with the objective increase in permeability (*r* = 0.250). Whether GI symptoms adequately reflect permeability and therefore can be used to guide maximal exertion while minimizing the risks of translocation warrant further investigation.

The authors recognize that this pilot study has limitations. The maximum T_CORE_ was limited to 39.7℃, which may not be high enough to demonstrate significant effects. Heatstroke is defined at a higher T_CORE_ (>40.0℃; Bouchama & Knochel, 2002), and, at in vitro temperatures exceeding this threshold, GI paracellular resistance falls proportionally to temperature increase (Dokladny et al., 2006). Furthermore, there is histological and functional evidence of increased GI permeability in vivo, at core temperatures above 41℃ (Lambert et al., 2002). In spite of this ethical limitation, the aims of the study were achieved as both trials elicited the same T_CORE_, exceeding the 39.0℃ threshold, which has been shown to “always” be associated with alterations in GI permeability (Pires et al., 2017). Based on this, comparisons between trials are appropriate even if they do not fully characterize the magnitude of GI permeability change. While exercise elicits a profound cardiovascular response, the study did not seek to identify a contributory role of neural involvement, which may be an important modulator of the response (Carabotti et al., 2015). The authors also acknowledge that this study does not include a control group or trial to measure baseline GI permeability, or measure changes after normothermic exercise; however, the study conditions between the two intervention trials in the present trial were replicated as tightly as possible, with each participant acting as their own comparator.

Future work should seek to quantify these responses against baseline/control L:R and data from a normothermic exercise trial, and investigate pharmacological and nutritional‐ or hydration‐related interventions to mitigate against heat stress. Strategies to reduce GI permeability, endotoxemia, and/or organ dysfunction have shown promising results via prior‐exercise glutamine ingestion (Pugh et al., 2017); mid‐exercise carbohydrate and protein intake (Snipe et al., 2017), and in animals, the administration of steroid (Walter & Gibson, 2020a) and antibiotics (Walter & Gibson, 2020b) following the development of exertional heat stress. This work should extend to a heterogeneous cohort of participants likely to be exposed to passive heat or EHI across a spectrum of thermal and exercise stressors. Finally, given the ability to measure plasma L:R in series more readily than urinary samples, future experiments should also consider the time‐course of responses during the onset and recovery from exercise and hyperthermia.

## 5 CONCLUSIONS

Gastrointestinal permeability, as measured by L:R disugar concentration in plasma, is increased more after exercise hyperthermia than by passive hyperthermia at a similar core temperature. The greater L:R ratio after exercise hyperthermia indicates that increased cardiovascular strain occurring during exercise hyperthermia, compared with passive hyperthermia, likely contributes to the increased GI permeability.

## CONFLICT OF INTEREST

The authors declare there are no competing interests.

## AUTHOR CONTRIBUTIONS

All authors designed the study and wrote the paper. All authors have seen and approved the final version. OG provided further statistical analysis. PW developed the method and performed the L:R analysis.

## Data Availability

The data that support the findings of this study are available from the corresponding author upon reasonable request.

## References

[phy214945-bib-0001] Badoer, E. (2010). Role of the hypothalamic PVN in the regulation of renal sympathetic nerve activity and blood flow during hyperthermia and in heart failure. American Journal of Physiology‐Renal Physiology, 298(4), F839–F846. 10.1152/ajprenal.00734.2009 20147365 PMC2853311

[phy214945-bib-0002] Borg, G. A. (1982). Psychophysical bases of perceived exertion. Medicine and Science in Sports and Exercise, 14, 377–381. 10.1249/00005768-198205000-00012 7154893

[phy214945-bib-0003] Bouchama, A., & Knochel, J. P. (2002). Heat stroke. New England Journal of Medicine, 346, 1978–1988. 10.1056/NEJMra011089 12075060

[phy214945-bib-0004] Carabotti, M., Scirocco, A., Maselli, M. A., & Severi, C. (2015). The gut‐brain axis: Interactions between enteric microbiota, central and enteric nervous systems. Annals of Gastroenterology, 28(2), 203–209. 25830558 PMC4367209

[phy214945-bib-0005] Dokladny, K., Moseley, P. L., & Ma, T. Y. (2006). Physiologically relevant increase in temperature causes an increase in intestinal epithelial tight junction permeability. The American Journal of Physiology: Gastrointestinal and Liver Physiology, 290, G204–G212. 10.1152/ajpgi.00401.2005 16407590

[phy214945-bib-0006] Fleming, S., Duncan, A., Russell, R., & Laker, M. (1996). Measurement of sugar probes in serum: An alternative to urine measurement in intestinal permeability testing. Clinical Chemistry, 42(3), 445–448. 10.1093/clinchem/42.3.445 8598111

[phy214945-bib-0007] González‐Alonso, J., Crandall, C. G., & Johnson, J. M. (2008). The cardiovascular challenge of exercising in the heat. The Journal of Physiology, 586(1), 45–53. 10.1113/jphysiol.2007.142158 17855754 PMC2375553

[phy214945-bib-0008] Hall, D. M., Buettner, G. R., Oberley, L. W., Xu, L., Matthes, R. D., & Gisolfi, C. V. (2001). Mechanisms of circulatory and intestinal barrier dysfunction during whole body hyperthermia. American Journal of Physiology‐Heart and Circulatory Physiology, 280(2), H509–H521. 11158946 10.1152/ajpheart.2001.280.2.H509

[phy214945-bib-0009] Hayashi, N., Yamaoka‐Endo, M., Someya, N., & Fukuba, Y. (2012). Blood flow in non‐muscle tissues and organs during exercise: Nature of splanchnic and ocular circulation. The Journal of Sports Medicine and Physical Fitness, 1(2), 281–286. 10.7600/jpfsm.1.281

[phy214945-bib-0010] James, C. A., Richardson, A. J., Watt, P. W., Gibson, O. R., & Maxwell, N. S. (2015). Physiological responses to incremental exercise in the heat following internal and external precooling. Scandinavian Journal of Medicine & Science in Sports, 25(Suppl. 1), 190–199. 10.1111/sms.12376 25943670

[phy214945-bib-0011] Jansen, G., Muskiet, F. A. J., Schierbeek, H., Berger, R., & Vanderslik, W. (1986). Capillary gas‐chromatographic profiling of urinary, plasma and erythrocyte sugars and polyols as their trimethylsilyl derivatives, preceded by a simple and rapid prepurification method. Clinica Chimica Acta, 157, 277–294. 10.1016/0009-8981(86)90303-7 3731489

[phy214945-bib-0012] Koch, F., Thom, U., Albrecht, E., Weikard, R., Nolte, W., Kuhla, B., & Kuehn, C. (2019). Heat stress directly impairs gut integrity and recruits distinct immune cell populations into the bovine intestine. Proceedings of the National Academy of Sciences of the United States of America, 116(21), 10333–10338. 10.1073/pnas.1820130116 31064871 PMC6535017

[phy214945-bib-0013] Lambert, G. P., Gisolfi, C. V., Berg, D. J., Moseley, P. L., Oberley, L. W., & Kregel, K. C. (2002). Selected contribution: Hyperthermia‐induced intestinal permeability and the role of oxidative and nitrosative stress. Journal of Applied Physiology, 92, 1750–1761. 10.1152/japplphysiol.00787.2001 11896046

[phy214945-bib-0014] Lim, C. L. (2018). Heat sepsis precedes heat toxicity in the pathophysiology of heat stroke – A new paradigm on an ancient disease. Antioxidants, 7(11), 149. 10.3390/antiox7110149 PMC6262330 30366410

[phy214945-bib-0015] March, D. S., Marchbank, T., Playford, R. J., Jones, A. W., Thatcher, R., & Davison, G. (2017). Intestinal fatty acid‐binding protein and gut permeability responses to exercise. European Journal of Applied Physiology, 117(5), 931–941. 10.1007/s00421-017-3582-4 28290057 PMC5388720

[phy214945-bib-0016] Nichols, A. W. (2014). Heat‐related illness in sports and exercise. Current Reviews in Musculoskeletal Medicine, 7(4), 355–365. 10.1007/s12178-014-9240-0 25240413 PMC4596225

[phy214945-bib-0017] Pease, S., Bouadma, L., Kermarrec, N., Schortgen, F., Regnier, B., & Wolff, M. (2009). Early organ dysfunction course, cooling time and outcome in classic heatstroke. Intensive Care Medicine, 35(8), 1454–1458. 10.1007/s00134-009-1500-x 19404610

[phy214945-bib-0018] Pires, W., Veneroso, C. E., Wanner, S. P., Pacheco, D. A. S., Vaz, G. C., Amorim, F. T., Tonoli, C., Soares, D. D., & Coimbra, C. C. (2017). Association between exercise‐induced hyperthermia and intestinal permeability: A systematic review. Sports Medicine, 47(7), 1389–1403. 10.1007/s40279-016-0654-2 27943148

[phy214945-bib-0019] Pugh, J. N., Sage, S., Hutson, M., Doran, D. A., Fleming, S. C., Highton, J., Morton, J. P., & Close, G. L. (2017). Glutamine supplementation reduces markers of intestinal permeability during running in the heat in a dose‐dependent manner. European Journal of Applied Physiology, 117, 2569–2577. 10.1007/s00421-017-3744-4 29058112 PMC5694515

[phy214945-bib-0020] Qamar, M. I., & Read, A. E. (1987). Effects of exercise on mesenteric blood flow in man. Gut, 28, 583–587. 10.1136/gut.28.5.583 3596339 PMC1432887

[phy214945-bib-0021] Sawka, M. N., Burke, L. M., Eichner, E. R., Maughan, R. J., Montain, S. J., & Stachenfeld, N. S. (2007). American College of Sports Medicine position stand. Exercise and fluid replacement. Medicine and Science in Sports and Exercise, 39(2), 377–390. 10.1249/mss.0b013e31802ca597 17277604

[phy214945-bib-0022] Shing, C. M., Peake, J. M., Lim, C. L., Briskey, D., Walsh, N. P., Fortes, M. B., Ahuja, K. D., & Vitetta, L. (2014). Effects of probiotics supplementation on gastrointestinal permeability, inflammation and exercise performance in the heat. European Journal of Applied Physiology, 114(1), 93–103. 10.1007/s00421-013-2748-y 24150782

[phy214945-bib-0023] Smetanka, R. D., Lambert, G. P., Murray, R., Eddy, D., Horn, M., & Gisolfi, C. V. (1999). Intestinal permeability in runners in the 1996 Chicago marathon. International Journal of Sport Nutrition and Exercise Metabolism, 9(4), 426–433. 10.1123/ijsn.9.4.426 10660873

[phy214945-bib-0024] Snipe, R. M. J., Khoo, A., Kitic, C. M., Gibson, P. R., & Costa, R. J. S. (2017). Carbohydrate and protein intake during exertional heat stress ameliorates intestinal epithelial injury and small intestine permeability. Applied Physiology, Nutrition, and Metabolism, 42(12), 1283–1292. 10.1139/apnm-2017-0361 28777927

[phy214945-bib-0025] Toner, M. M., Drolet, L. L., & Pandolf, K. B. (1986). Perceptual and physiological responses during exercise in cool and cold water. Perceptual and Motor Skills, 62, 211–220. 10.2466/pms.1986.62.1.211 3960662

[phy214945-bib-0026] Walter, E. J., & Gibson, O. R. (2020a). The efficacy of steroids in reducing morbidity and mortality from extreme hyperthermia and heatstroke – A systematic review. Pharmacology Research & Perspectives, 8(4), e00626. 10.1002/prp2.626 32666709 PMC7360483

[phy214945-bib-0027] Walter, E. J., & Gibson, O. R. (2020b). The efficacy of antibiotics in reducing morbidity and mortality from heatstroke – A systematic review. Journal of Thermal Biology, 88, 102509. 10.1002/prp2.626 32125990

[phy214945-bib-0028] Walter, E., Gibson, O. R., Stacey, M., Hill, N., Parsons, I. T., & Woods, D. (2021). Changes in gastrointestinal cell integrity after marathon running and exercise‐associated collapse. European Journal of Applied Physiology, 121(4), 1179–1187. 10.1007/s00421-021-04603-w 33512586

[phy214945-bib-0029] Walter, E. J., Hanna‐Jumma, S., Carraretto, M., & Forni, L. (2016). The pathophysiological basis and consequences of fever. Critical Care, 20, 200. 10.1186/s13054-016-1375-5 27411542 PMC4944485

[phy214945-bib-0030] Wilson, P. B. (2017). Frequency of chronic gastrointestinal distress in runners: Validity and reliability of a retrospective questionnaire. International Journal of Sport Nutrition and Exercise Metabolism, 27(4), 370–376. 10.1123/ijsnem.2016-0305 28253039

[phy214945-bib-0031] Zhang, H., Huizenga, C., Arens, E., & Wang, D. (2004). Thermal sensation and comfort in transient non‐uniform thermal environments. European Journal of Applied Physiology, 92, 728–733. 10.1007/s00421-004-1137-y 15221406

[phy214945-bib-0032] Zuhl, M. N., Lanphere, K. R., Kravitz, L., Mermier, C. M., Schneider, S., Dokladny, K., & Moseley, P. L. (2014). Effects of oral glutamine supplementation on exercise‐induced gastrointestinal permeability and tight junction protein expression. J Appl Physiol., 116, 183–191. 24285149 10.1152/japplphysiol.00646.2013 PMC3921361

